# Supplementary material for: Multi-modal cryo-EM reveals trimers of protein A10 to form the palisade layer in poxvirus cores
Source: Nat Struct Mol Biol. 2024 Feb 5;31(7):1114–23. doi: 10.1038/s41594-023-01201-6 (PMC11257981; doi:10.1038/s41594-023-01201-6)
Supplement: Supplementary file 1 — Supplementary Figures 1 and 2, Supplementary Tables 1 and 2, and legends for Supplementary Videos 1–4 [file 41594_2023_1201_MOESM1_ESM.pdf]

nature structural & molecular biology

Article

https://doi.org/10.1038/s41594-023-01201-6

Multi-modal cryo-EM reveals trimers of protein A10 to form the palisade layer in poxvirus cores

In the format provided by the authors and unedited

Supplementary Figures

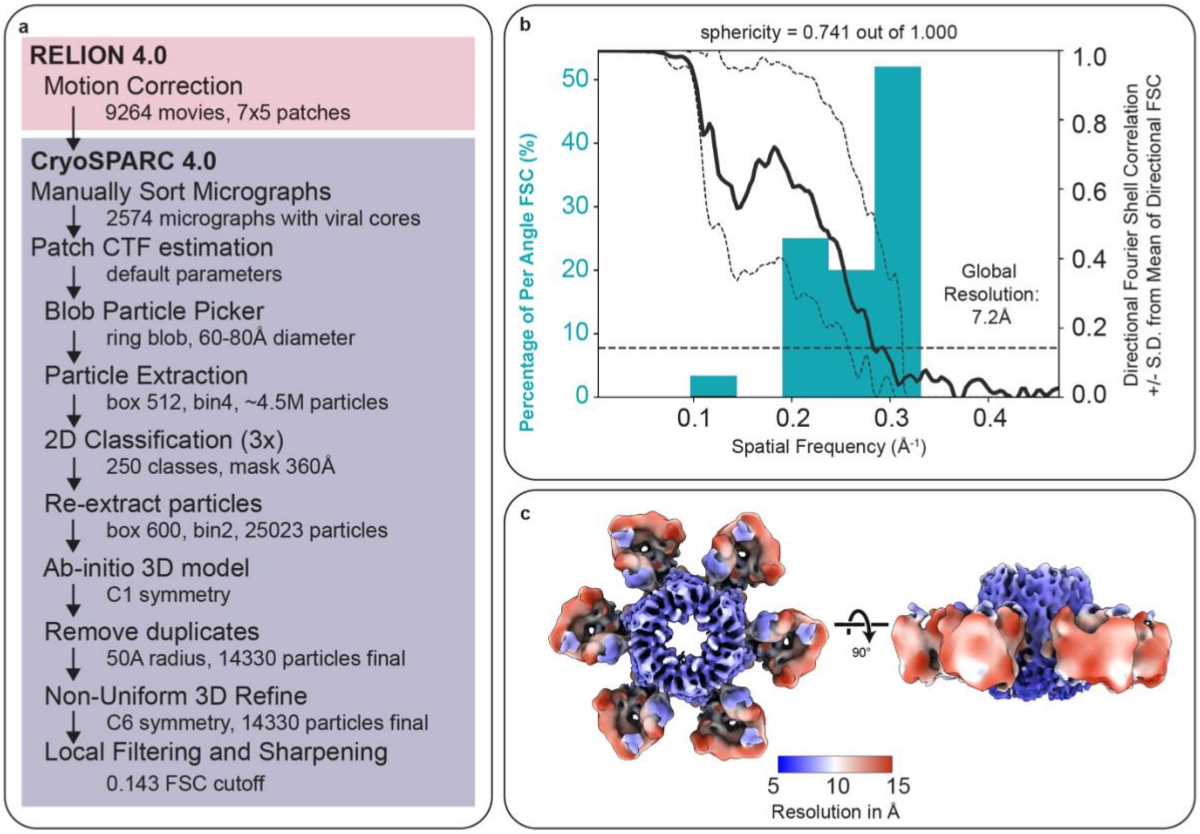

Supplementary Figure 1: SPA processing workflow of the flower-shaped pore

a) Summary of processing steps used in the SPA workflow. **b)** 3D FSC calculations of the masked CryoSPARC half maps. The cyan histogram depicts the fraction of particles that reach the corresponding resolution, and the black curve shows the global FSC +/- SD of FSCs calculated with extensive angular sampling. The global resolution indicated is at FSC 0.143 cutoff. **c)** Local resolution of the flower-shaped pore cryo-EM density map.

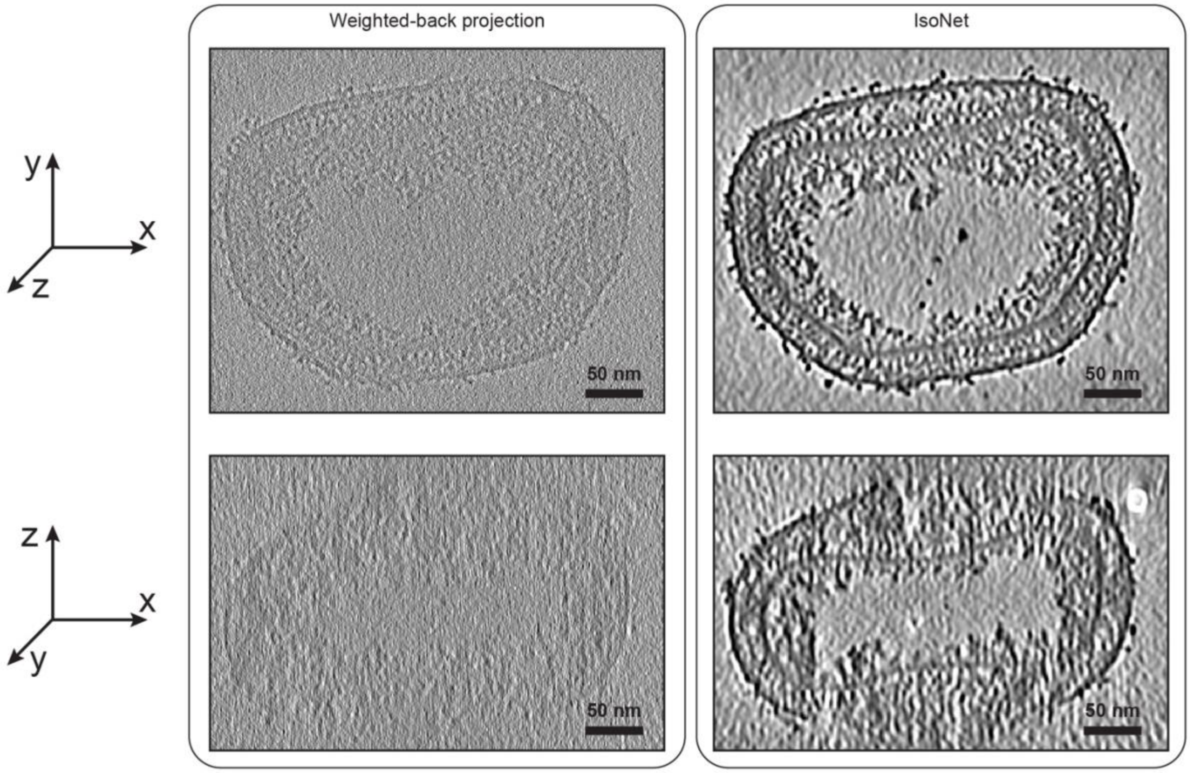

Supplementary Figure 2: Comparison of weighted-back-projected (WBP) tomogram and IsoNet-corrected tomogram of VACV mature virions

The view axis of the individual slices of the same tomogram are referenced with a coordinate system on the left, showing the viewing direction onto the xy and xz plane on top and bottom, respectively. The tomogram shown here is identical to the one shown in Figure 1B and is representative for 15 tomograms.

Supplementary Tables

UniProt ID	Common Names	Genes	Sequence Coverage %	Peptide Counts	Log 10 expression
P16715	*Major core protein A10 (4a) precursor*	*VACWR129*	60.5	69	9.5060
P03295	*Core protein L4 (VP8)*	*VACWR091*	54.2	16	9.1462
P26669	*Cu-Zn superoxide dismutase-like protein A45R*	*VACWR171*	91.2	9	8.8556
P04195	*Cell surface-binding protein*	*VACWR113*	53.9	21	8.7152
P07396	*Phosphoprotein F17*	*VACWR056*	32.7	6	8.6891
P29191	*A4 (39kDa) core protein*	*VACWR123*	17.8	4	8.2534
P04298	*mRNA-capping enzyme catalytic subunit*	*VACWR106*	62.8	56	8.2447
P68692	*Glutaredoxin-1*	*VACWR069*	24.1	4	8.2045
P06440	*Major core protein A3 (4b)*	*VACWR122*	34.4	17	8.1866
Q76ZN5	*Profilin*	*VACWR167*	39.8	6	8.0401
P12926	*Core protease I7*	*VACWR076*	24.3	11	7.8991
P07614	*Protein L3*	*VACWR090*	32.9	12	7.8896
P04318	*mRNA-capping enzyme regulatory subunit*	*VACWR117*	72.1	19	7.8743
P07242	*Late transcription elongation factor H5*	*VACWR103*	44.8	10	7.8030
P24758	*Protein A26*	*VACWR149*	35	15	7.7764
P07616	*Protein J1*	*VACWR093*	19.6	4	7.7718
P18377	*Phospholipase-D-like protein K4*	*VACWR035*	30.4	15	7.7231
P68623	*Protein L5*	*VACWR092*	24.2	4	7.6414
P07611	*Myristoylated protein G9*	*VACWR087*	26.5	8	7.4532
P68710	*DNA polymerase processivity factor component A20*	*VACWR141*	1.6	1	7.4508

Supplementary Table 1: Mass spectrometry of components from isolated cores
List of mass spectrometry results from the soluble fraction core sample, filtered to exclusively show proteins encoded by VACV. Please note that the precursor proteins are also listed using the UniProt annotation, due to the way of how proteins have been identified computationally within our proteomics data.

A10 (UniProt ID/residue range/UniProt name)	23K UniProt ID/residue range/UniProt name)	A3 UniProt ID/residue range/UniProt name)	L4 UniProt ID/residue range/UniProt name)	A4 (UniProt ID/residue range/UniProt name)
D5FLF11_614/Orthopoxvirus	D5FLF1/698_891/Orthopoxvirus	A0A223FMU0/62_641/Centapoxvirus	A0A223FMU1/33_250/Murmansk_poxvirus	P0DOP1/1_271/Orthopoxvirus
A0A223FMW2/1_621/Centapoxvirus	G3EI04/705_897/Yokapox_virus	G3EHZ6/62_646/Yokapox_virus	G3EIE9/33_251/Centapoxvirus	A0A5B8XAC6/1_293/Alaskapox_virus
G3EI04/1_616/Yokapox_virus	A0A223FMW2/711_903/Centapoxvirus	Q08FP9/78_652/Cervidpoxvirus	A0A2I6BQS5/33_250/Cervidpoxvirus	A0A2I6J1D1/1_211/Vaccinia_virus
A0A514TRC3/1_612/Hypsugopox_virus	Q9DHL2/709_902/Yatapoxvirus	Q8V3K5/76_652/Swinepox_virus	Q9DHQ0/33_247/Yatapoxvirus	A0A0G3G2L5/1_289/Raccoon_poxvirus
R4JS96/1_612/Eptesipox_virus	A0A2I6BQV3/722_915/Cervidpoxvirus	Q9DHL9/73_657/Yatapoxvirus	H6TA55/35_251/Oryzopoxvirus	A0A1C9KBU4/1_303/Orthopoxvirus
Q9DHL2/3_614/Yatapoxvirus	A0A2U8U5V7/712_904/Baiomys_poxvirus	A0A2H4EUE8/93_661/Capripoxvirus	Q8V3N4/34_251/Swinepox_virus	
A0A2I6BQV3/1_614/Cervidpoxvirus	Q8V3J8/710_904/Swinepox_virus	Q9Q8Y6/90_653/Leporipoxvirus	A0A7D0UBN6/33_251/Brazilian_porcupinepox_virus_1	
Q8V3J8/1_614/Swinepox_virus	Q98280/698_889/Molluscum_contagiosum_virus	A0A7D0Q8N2/87_648/Brazilian_porcupinepox_virus_1	A0A1C9HHS7/33_251/Capripoxvirus	
A0A1B3B680/1_616/Capripoxvirus	A0A7D0UCR1/707_899/Brazilian_porcupinepox_virus_1	U3UBJ5/88_661/Squirrelpox_virus	A0A220T6C5/33_252/Poxviridae	
Q9Q8X9/1_618/Leporipoxvirus	A0A1B3B680/710_903/Capripoxvirus	H6TA83/79_651/Oryzopoxvirus	K4JL49/33_249/Leporipoxvirus	
H6TA89/1_615/Oryzopoxvirus	A0A2C9DT77/701_893/Macropopoxvirus	A0A220T6G3/69_663/4Eptesipox_virus	A0A6B9R326/37_254/Cetacean_poxvirus_1	
A0A2U8U5V7/1_606/Baiomys_poxvirus	H6TA89/706_898/Oryzopoxvirus	A0A6B9R356/89_649/Cetacean_poxvirus_1	A0A1B1MRG1/34_253/Pteropox_virus	
A0A7D0UCR1/1_613/Brazilian_porcupinepox_virus_1	A0A650AJ19/692_884/Equine_molluscum_contagiosum-like_virus	A0A514TRC0/67_666/Hypsugopox_virus	U3UBJ3/34_249/Squirrelpox_virus	
U3UBK3/1_605/Squirrelpox_virus	U3UBK3/709_899/Squirrelpox_virus	A0A7G5AXA7/105_674/Molluscum_contagiosum_virus	A0A0R8I369/33_250/Parapoxvirus	
A0A6B9R265/1_599/Cetacean_poxvirus_1	Q9Q8X9/708_902/Leporipoxvirus	A0A650AJ10/80_669/Equine_molluscum_contagiosum-like_virus	A0A2U9QHN1/36_253/Sea_otter_poxvirus	
A0A1B1MRH6/3_605/Pteropox_virus	R4JS96/713_908/Eptesipox_virus	A0A2C9DT70/121_708/Macropopoxvirus	A0A1Z3GCV2/33_249/Seal_parapoxvirus	
Q98280/1_601/Molluscum_contagiosum_virus	A0A514TRC3/715_910/Hypsugopox_virus	A0A1B1MRG6/105_665/Pteropox_virus	Q6TVD8/32_248/Bovine_papular_stomatitis_virus	
A0A2U9QHR8/1_602/Sea_otter_poxvirus	A0A6B9R265/707_898/Cetacean_poxvirus_1	A0A2U9QHT6/76_659/Sea_otter_poxvirus	A0A0A7MES2/33_250/Parapoxvirus_red_deer/HL953	
A0A650AJ19/1_601/Equine_molluscum_contagiosum-like_virus	A0A2U9QHR8/722_913/Sea_otter_poxvirus	A0A0M3PB61/81_655/Avipoxvirus	A0A7G5AX74/31_249/Molluscum_contagiosum_virus	
A0A6G8HJ41/1_604/Poxviridae_sp.	A0A6G8HJ41/726_919/Poxviridae_sp.	A0A1V0QGY3/82_659/Poxviridae	A0A650AJD5/31_246/Equine_molluscum_contagiosum-like_virus	
A0A1Z3GCS2/9_597/Seal_parapoxvirus	A0A895A117/697_888/Teiidae_poxvirus_1	A0A894ZUL5/75_647/Teiidae_poxvirus_1	A0A2C9DT39/31_246/Macropopoxvirus	
A0A2C9DT77/1_607/Macropopoxvirus	A0A1Z3GCS2/712_905/Seal_parapoxvirus	A0A649Z203/89_658/Poxviridae	A0A895A365/32_249/Teiidae_poxvirus_1	
A0A0R8I2Z9/9_602/Parapoxvirus	Q9J559/700_891/Avipoxvirus	P17355/72_656/Avipoxvirus	Q6VZH1/31_249/Poxviridae	
A0A0A7MC58/9_604/Parapoxvirus_red_deer/HL953	A0A1B1MRH6/728_919/Pteropox_virus	A0A1Z3GCS5/99_676/Seal_parapoxvirus	A0A0M3ZEM5/32_251/Turkeypox_virus	
A0A895A117/1_603/Teiidae_poxvirus_1	A0A0M3ZPP0/711_899/Turkeypox_virus	Q070C7/110_674/Crocodylidpoxvirus		
A0A890UVV5/1_604/Cheloniid_poxvirus_1	Q6TVA2/716_908/Bovine_papular_stomatitis_virus	A0A0R8HJ36/97_675/Parapoxvirus		
Q6TVA2/10_603/Bovine_papular_stomatitis_virus	A0A0R8I2Z9/713_905/Parapoxvirus	Q6TVA9/99_680/Bovine_papular_stomatitis_virus		
Q9J559/1_604/Avipoxvirus	A0A0A7MC58/717_909/Parapoxvirus_red_deer/HL953	D3IZM7/99_676/Parapoxvirus		
Q6VZA0/1_604/Poxviridae	A0A890UVV5/701_893/Cheloniid_poxvirus_1	A0A0A7M9S5/123_686/Parapoxvirus_red_deer/HL953		
A0A1V0S839/2_605/Shearwaterpox_virus	Q6VZA0/701_893/Poxviridae	D3GH01/3_445/Canadian_squirrelpox_virus		
A0A0M3ZPP0/1_608/Turkeypox_virus	A0A1V0S839/705_897/Shearwaterpox_virus			

Supplementary Table 2: Sequences included in the MSA-alignment for Consurf analysis

Sequences for Consurf analysis for A10, 23K, A3, L4 and A4 are ordered based on MSA-alignment E-value.

Supplementary Movies

Supplementary Video 1: Video of a cryo-electron tomogram containing an intact purified MV. The shown tomogram corresponds to the tomogram shown in Figure 1b.
Supplementary Video 2: Video of a cryo-electron tomogram containing an isolated core. The shown tomogram corresponds to the tomogram shown in Figure 1c.
Supplementary Video 3: Morph between the AlphaFold-predicted A10 trimer, and the refined A10 trimer based on our EM reconstruction
Supplementary Video 4: Video showing the interactions stabilizing the A10 trimer as described in Extended Data Figure 6.
