# Supplementary material for: Multi-modal cryo-EM reveals trimers of protein A10 to form the palisade layer in poxvirus cores
Source: Nat Struct Mol Biol. 2024 Feb 5;31(7):1114–23. doi: 10.1038/s41594-023-01201-6 (PMC11257981; doi:10.1038/s41594-023-01201-6)
Supplement: Supplementary file 2 — Reporting Summary [file 41594_2023_1201_MOESM2_ESM.pdf]

nature portfolio

Corresponding author(s): Florian KM Schur

Last updated by author(s): Dec 4, 2023

Reporting Summary

Nature Portfolio wishes to improve the reproducibility of the work that we publish. This form provides structure for consistency and transparency in reporting. For further information on Nature Portfolio policies, see our Editorial Policies and the Editorial Policy Checklist.

Statistics

For all statistical analyses, confirm that the following items are present in the figure legend, table legend, main text, or Methods section.

n/a	Confirmed	
☐	☒	The exact sample size (*n*) for each experimental group/condition, given as a discrete number and unit of measurement
☒	☐	A statement on whether measurements were taken from distinct samples or whether the same sample was measured repeatedly
☒	☐	The statistical test(s) used AND whether they are one- or two-sided<br>*Only common tests should be described solely by name; describe more complex techniques in the Methods section.*
☒	☐	A description of all covariates tested
☒	☐	A description of any assumptions or corrections, such as tests of normality and adjustment for multiple comparisons
☒	☐	A full description of the statistical parameters including central tendency (e.g. means) or other basic estimates (e.g. regression coefficient) AND variation (e.g. standard deviation) or associated estimates of uncertainty (e.g. confidence intervals)
☒	☐	For null hypothesis testing, the test statistic (e.g. *F*, *t*, *r*) with confidence intervals, effect sizes, degrees of freedom and *P* value noted<br>*Give P values as exact values whenever suitable.*
☒	☐	For Bayesian analysis, information on the choice of priors and Markov chain Monte Carlo settings
☒	☐	For hierarchical and complex designs, identification of the appropriate level for tests and full reporting of outcomes
☒	☐	Estimates of effect sizes (e.g. Cohen's *d*, Pearson's *r*), indicating how they were calculated

Our web collection on statistics for biologists contains articles on many of the points above.

Software and code

Policy information about availability of computer code

Data collection	Cryo-electron tomography data was collected with the SerialEM software package version 3.8.<br>DigitalMicrograph 3.4.3 as integrated into the Gatan Microscopy Suite v3.3 (Gatan) was used for energy filter tuning.<br>Automated single particle cryo-EM data was collected using EPU version 2.13 (Thermo Fisher Scientific) or SerialEM software package version 4.0.
Data analysis	Tomoman (08042020) was used to sort and create stacks.<br>Defocus was estimated using CTFFIND4 4.1.14.<br>IMOD (version 4.9.12) was used for tilt series alignment and to generate binned tomograms with weighted back projection.<br>The full tomograms were reconstructed in NovaCTF (version from 2018).<br>Missing wedge was corrected using Isonet 0.2.<br>Subtomogram averaging was performed in Dynamo version 1.1.333.<br>Single particle cryo-EM data were motion-corrected using the RELION 4.0-beta2 implementation of MotionCorr2 (version 4.0-beta2).<br>Single particle cryo-EM data were further processed using cryoSPARC 4.0.0 and RELION 4.0.<br>Volume projections were generated using EMAN version 1.9.<br>3D FSC calculations were done using the Salk Remote 3DFSC processing server.<br>Protein structures were computationally predicted using Alphafold 2.3.2 and Colabfold 1.5.2.<br>Atomic models were relaxed into cryo-EM density using Rosetta version 3.12.<br>Atomic model was manually inspected using Coot 0.8.9.1.<br>UCSF Chimera version 1.17.1 was used for symmetrization of atomic models.<br>UCSF ChimeraX version 1.5 was used for RMSD calculations and visualization.<br>Structural similarity analysis of Vaccinia virus core proteins was performed using Foldseek version 7-04e0ec8 (https://search.foldseek.com/

search) and Dali (accessed online August 2023) (http://ekhidna2.biocenter.helsinki.fi/dali/).
Conservation analysis was performed using ConSurf (accessed online September 2023) (https://consurf.tau.ac.il/).
Phenix version 1.20-dev-4224 was used for density modification.
Protein topology diagrams were prepared using Pro-Origami (version 1.0).
Adobe Premiere Pro 2023 was used for movie generation.
Adobe Illustrator 2023 was used for making figures.
Mass Spec data was analyzed in DIANN software version 1.8.1.

For manuscripts utilizing custom algorithms or software that are central to the research but not yet described in published literature, software must be made available to editors and reviewers. We strongly encourage code deposition in a community repository (e.g. GitHub). See the Nature Portfolio guidelines for submitting code & software for further information.

Data

Policy information about availability of data

All manuscripts must include a data availability statement. This statement should provide the following information, where applicable:

- Accession codes, unique identifiers, or web links for publicly available datasets
- A description of any restrictions on data availability
- For clinical datasets or third party data, please ensure that the statement adheres to our policy

The electron microscopy density maps of the A10 trimer and the hexameric flower-shaped pore, the subtomogram average of the palisade layer, as well as representative tomograms for complete viruses as well as isolated cores have been deposited in the Electron Microscopy Data Bank under accession codes: EMD-17410, EMD-17411, EMD-17412, EMD-17413, EMD-17414 and EMD-18452.
The refined model of the A10 trimer has been deposited in the Protein Data Bank accession code: PDB 8P4K.
The UniProt codes of VACV core proteins used for structure prediction are: A10 (P16715), A3 (P06440), A4 (P29191), L4 (P03295).
GenBank Protein IDs of Variola virus A10 (ABF23487.1), Monkeypox virus A10 (YP_010377118.1), Rabbipox virus A10 (AAS49831.1), Cowpox virus A10 (ADZ29251.1), Ectromelia virus A10 (NP_671631.1), Orf virus P4a (AY386264.1), Amsacta moorei entomopoxvirus AMV139 (NP_064921.1), Melanoplus sanguinipes entomopoxvirus putative core protein P4a homolog (AF063866.1) were used for protein sequence alignment.
Source data for Mass Spectometry experiments is provided as zip-archived Supplementary Information with this manuscript

Research involving human participants, their data, or biological material

Policy information about studies with human participants or human data. See also policy information about sex, gender (identity/presentation), and sexual orientation and race, ethnicity and racism.

Reporting on sex and gender	n/a
Reporting on race, ethnicity, or other socially relevant groupings	n/a
Population characteristics	n/a
Recruitment	n/a
Ethics oversight	n/a

Note that full information on the approval of the study protocol must also be provided in the manuscript.

Field-specific reporting

Please select the one below that is the best fit for your research. If you are not sure, read the appropriate sections before making your selection.

☒ Life sciences ☐ Behavioural & social sciences ☐ Ecological, evolutionary & environmental sciences

For a reference copy of the document with all sections, see nature.com/documents/nr-reporting-summary-flat.pdf

Life sciences study design

All studies must disclose on these points even when the disclosure is negative.

Sample size	9264 micrographs were collected for single particle analysis, which was sufficient to generate a high resolution density map for fitting of an atomic model. We used 15 tilt-series for subtomogram averaging, which was sufficient to generate a subtomogram average showing structural features of relevance.
Data exclusions	As is customary in cryo-EM data processing, entire micrographs were discarded based on poor quality metrics such as blur (poor CTF fit) or contamination. From the subset of retained micrographs, individual particles were discarded based on standard processing pipelines of 2D and 3D classification to remove particles which did not contribute valuable high resolution information.
Replication	Replication is not performed in macromolecular structural studies. Methods established in the field of structural biology were performed to

Replication	ensure validity of the study.
Randomization	Randomization of samples was not relevant in this study since structural characterization was exploratory and no grouping was necessary in order to make relevant comparisons or to draw conclusions.
Blinding	Blinding was not relevant in this study since macromolecular structures and assembly details were unknown to any researcher involved.

Reporting for specific materials, systems and methods

We require information from authors about some types of materials, experimental systems and methods used in many studies. Here, indicate whether each material, system or method listed is relevant to your study. If you are not sure if a list item applies to your research, read the appropriate section before selecting a response.

Materials & experimental systems

n/a	Involved in the study
☒	☐ Antibodies
☐	☒ Eukaryotic cell lines
☒	☐ Palaeontology and archaeology
☒	☐ Animals and other organisms
☒	☐ Clinical data
☒	☐ Dual use research of concern
☒	☐ Plants

Methods

n/a	Involved in the study
☒	☐ ChIP-seq
☒	☐ Flow cytometry
☒	☐ MRI-based neuroimaging

Eukaryotic cell lines

Policy information about cell lines and Sex and Gender in Research

Cell line source(s)	HeLa cells were kindly provided by the lab of Michael Sixt at ISTA, which were initially obtained from ATCC.
Authentication	not performed
Mycoplasma contamination	Cells were tested negative for mycoplasma contamination.
Commonly misidentified lines (See ICLAC register)	none
