# Supplementary material for: Multi-modal cryo-EM reveals trimers of protein A10 to form the palisade layer in poxvirus cores
Source: Nat Struct Mol Biol. 2024 Feb 5;31(7):1114–23. doi: 10.1038/s41594-023-01201-6 (PMC11257981; doi:10.1038/s41594-023-01201-6)
Supplement: Supplementary file 9 — DiaNN-identification results, associated with results in Supplementary Table 1 [file 41594_2023_1201_MOESM9_ESM.zip › SupplementaryData_2/report.pdf]

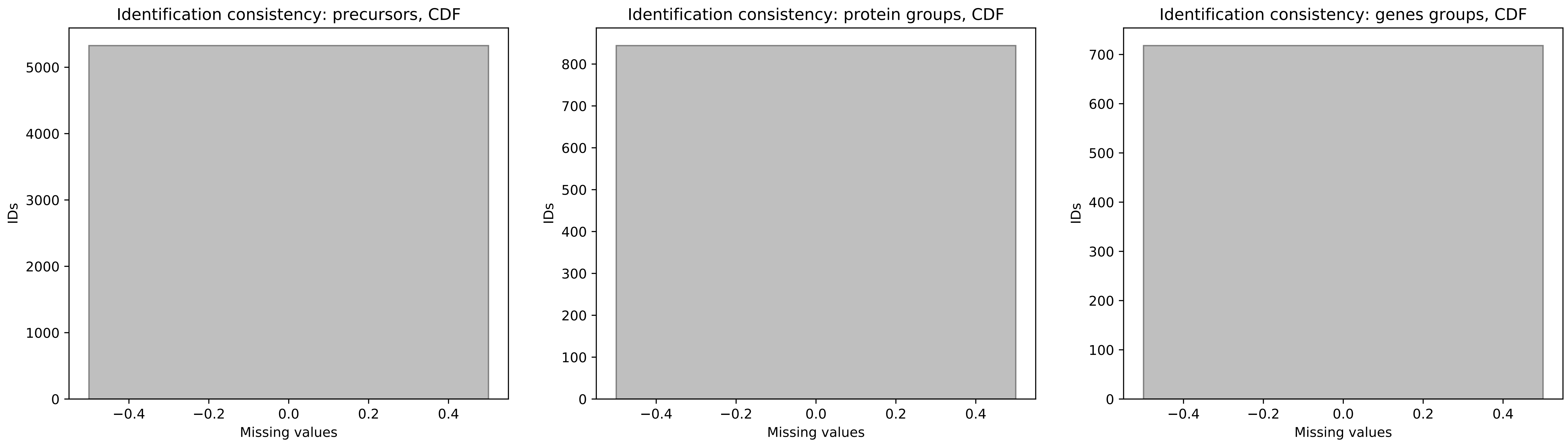

Identification consistency: precursors, CDF
IDs
Missing values
Identification consistency: protein groups, CDF
IDs
Missing values
Identification consistency: genes groups, CDF
IDs
Missing values

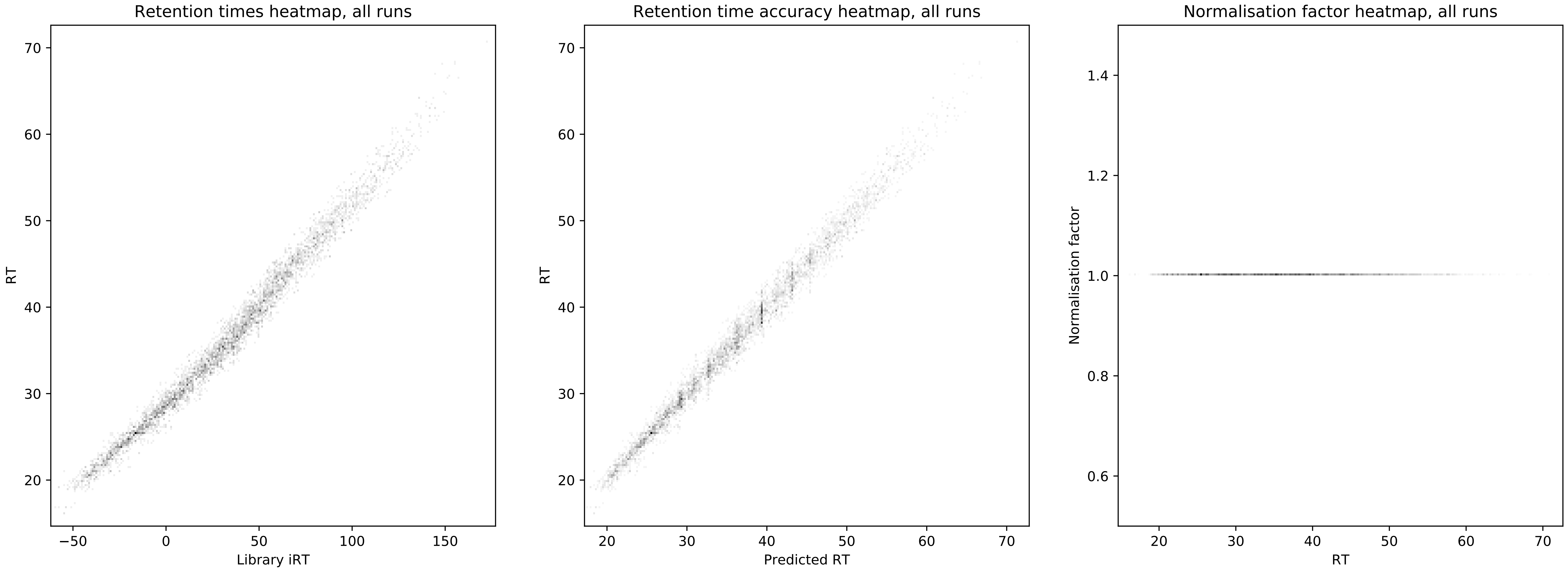
Retention times heatmap, all runs
RT
Library iRT
Retention time accuracy heatmap, all runs
RT
Predicted RT
Normalisation factor heatmap, all runs
Normalisation factor
RT

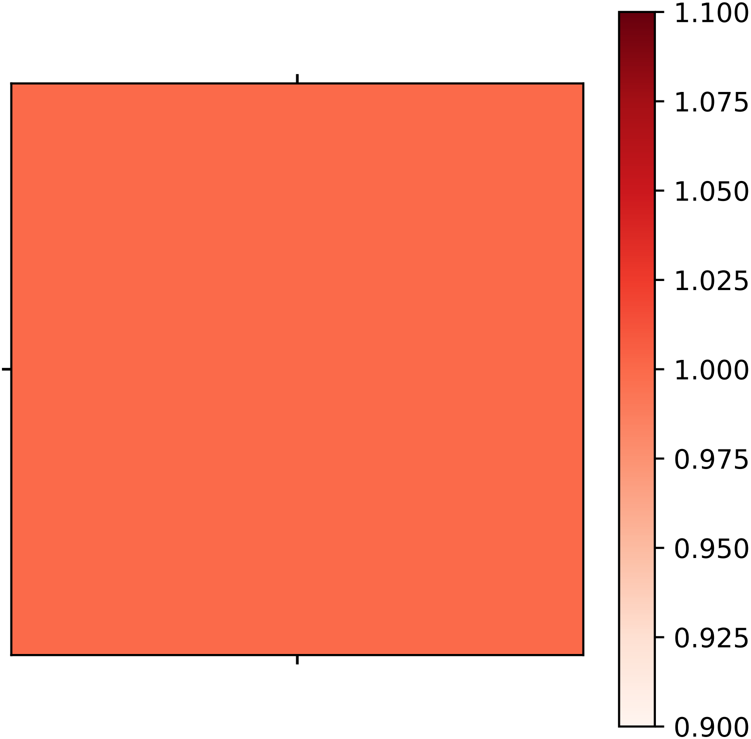
1.100
1.075
1.050
1.025
1.000
0.975
0.950
0.925
0.900

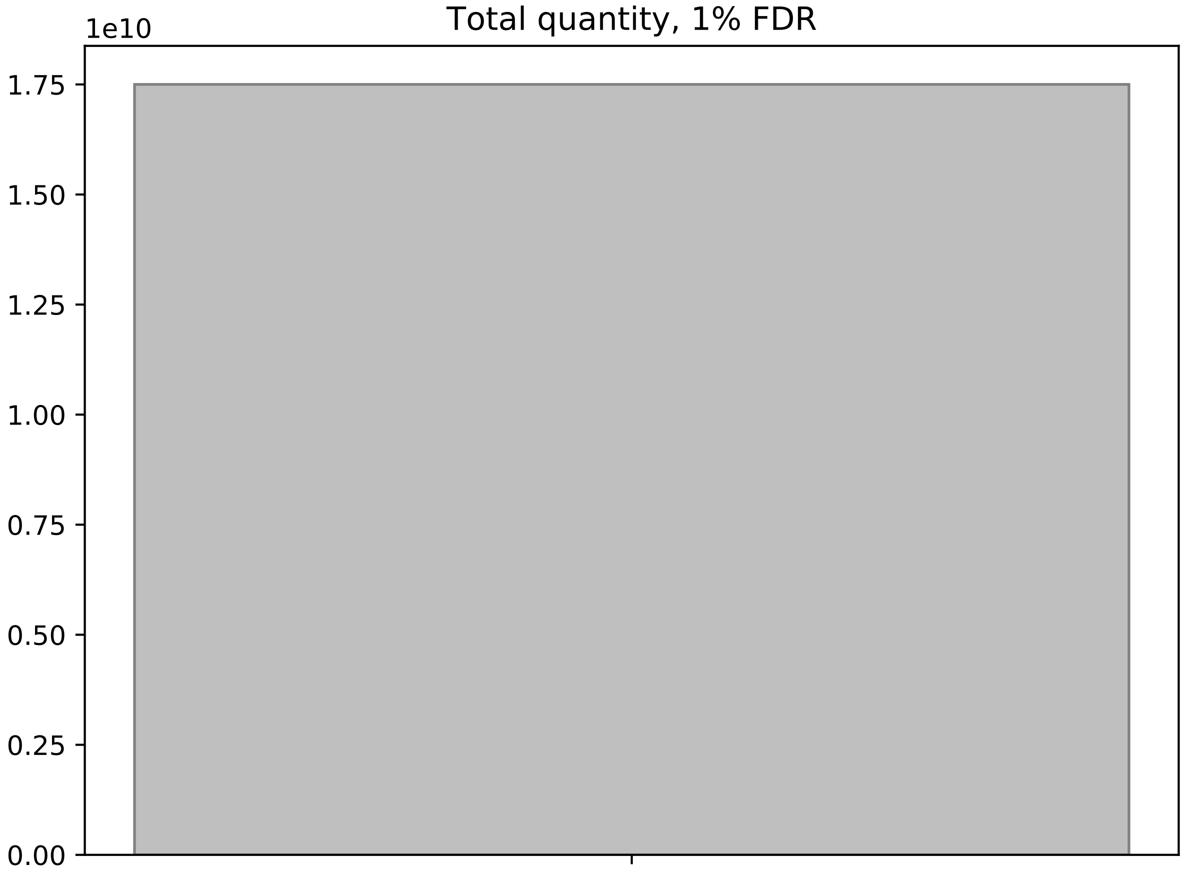

Total quantity, 1% FDR
1e10
1.75
1.50
1.25
1.00
0.75
0.50
0.25
0.00

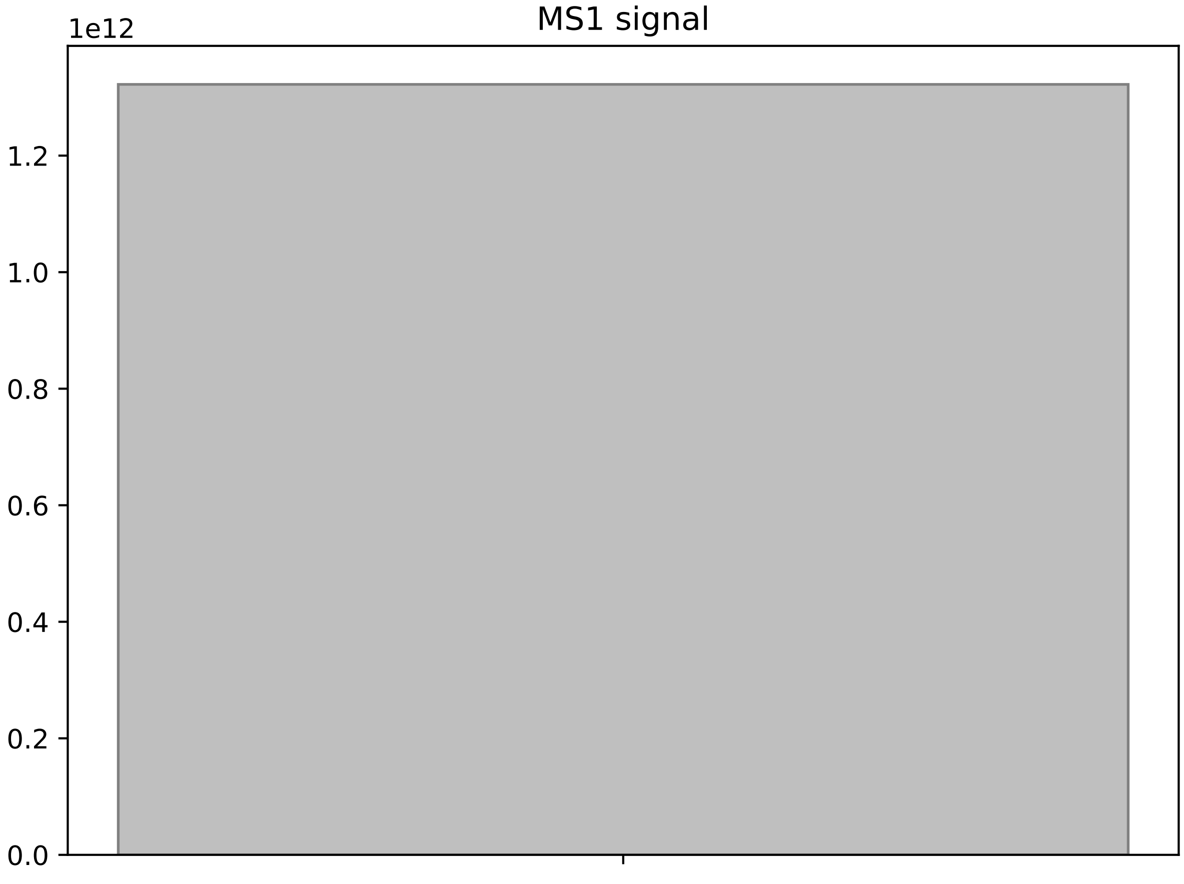

MS1 signal
1e12
1.2
1.0
0.8
0.6
0.4
0.2
0.0

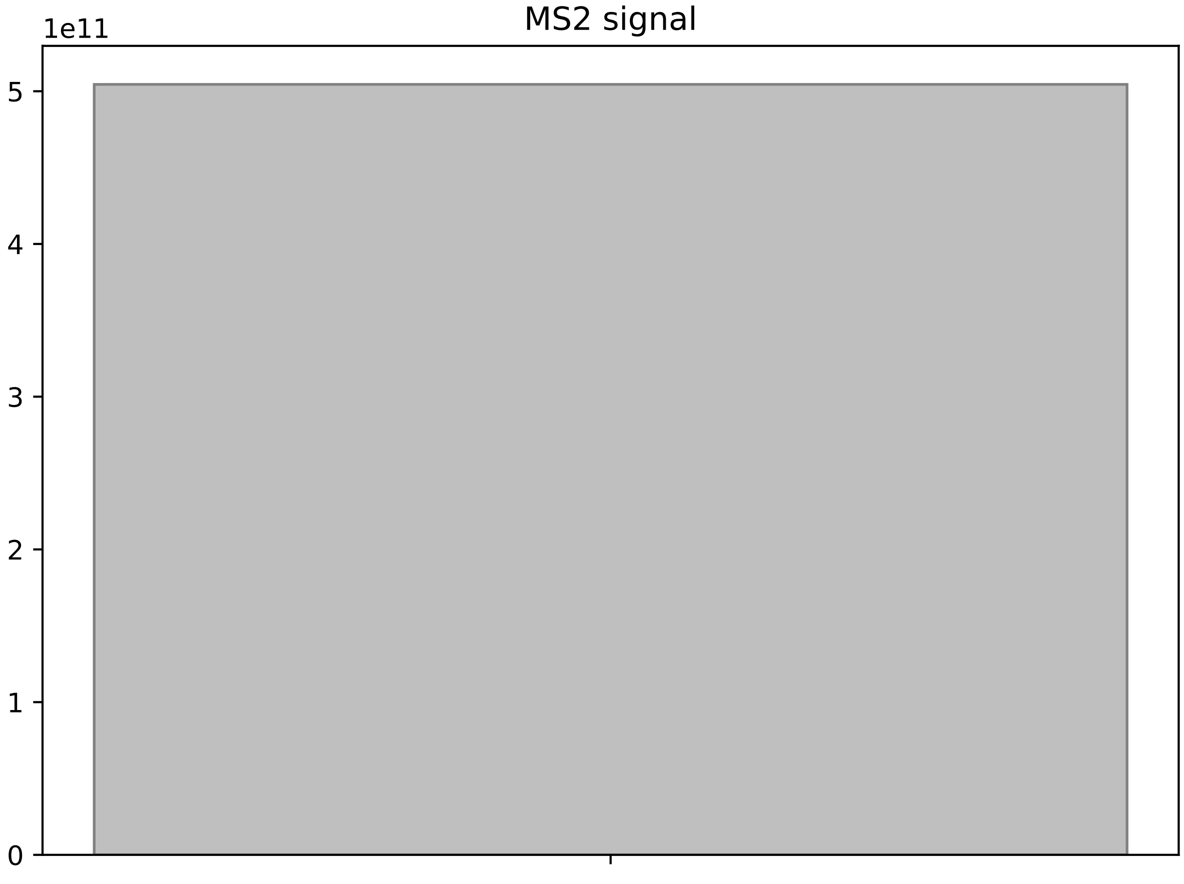
MS2 signal
1e11
5
4
3
2
1
0

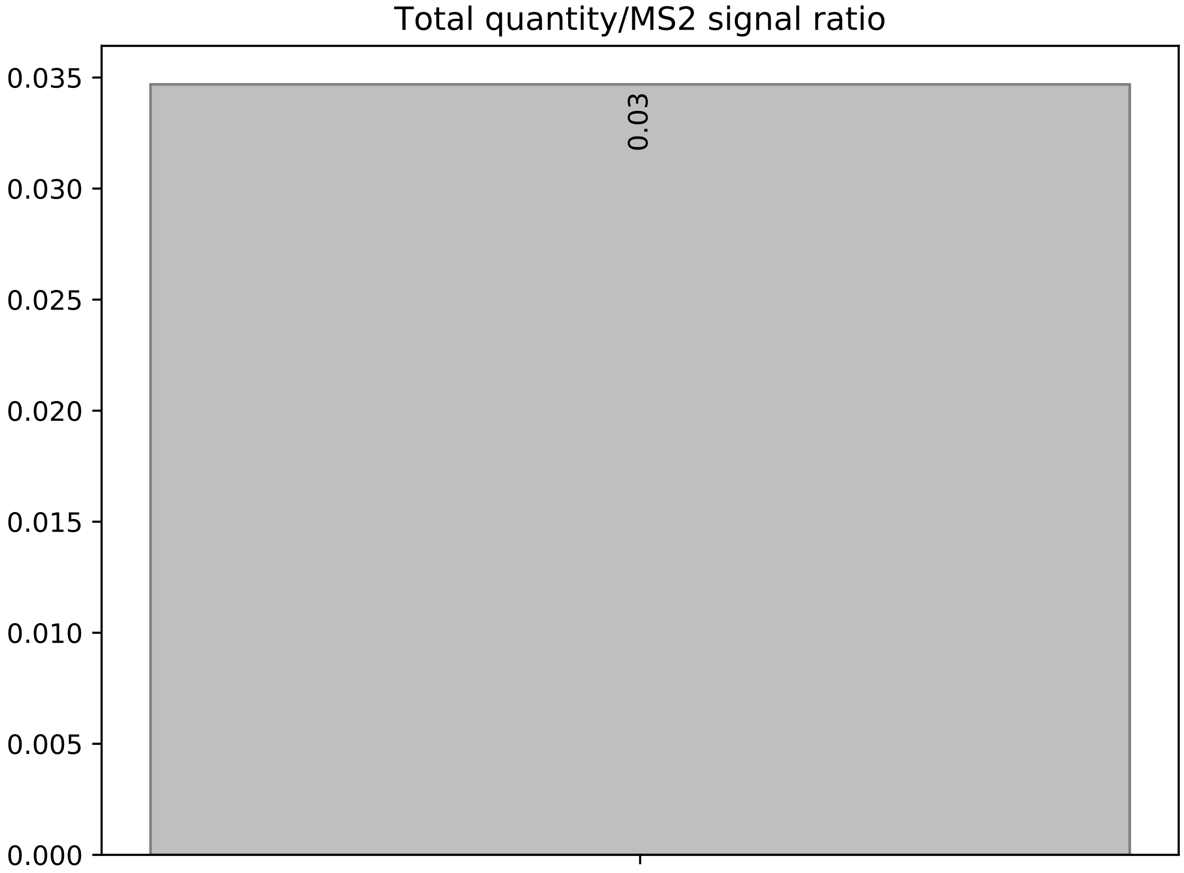
Total quantity/MS2 signal ratio
0.035
0.030
0.025
0.020
0.015
0.010
0.005
0.000
0.03

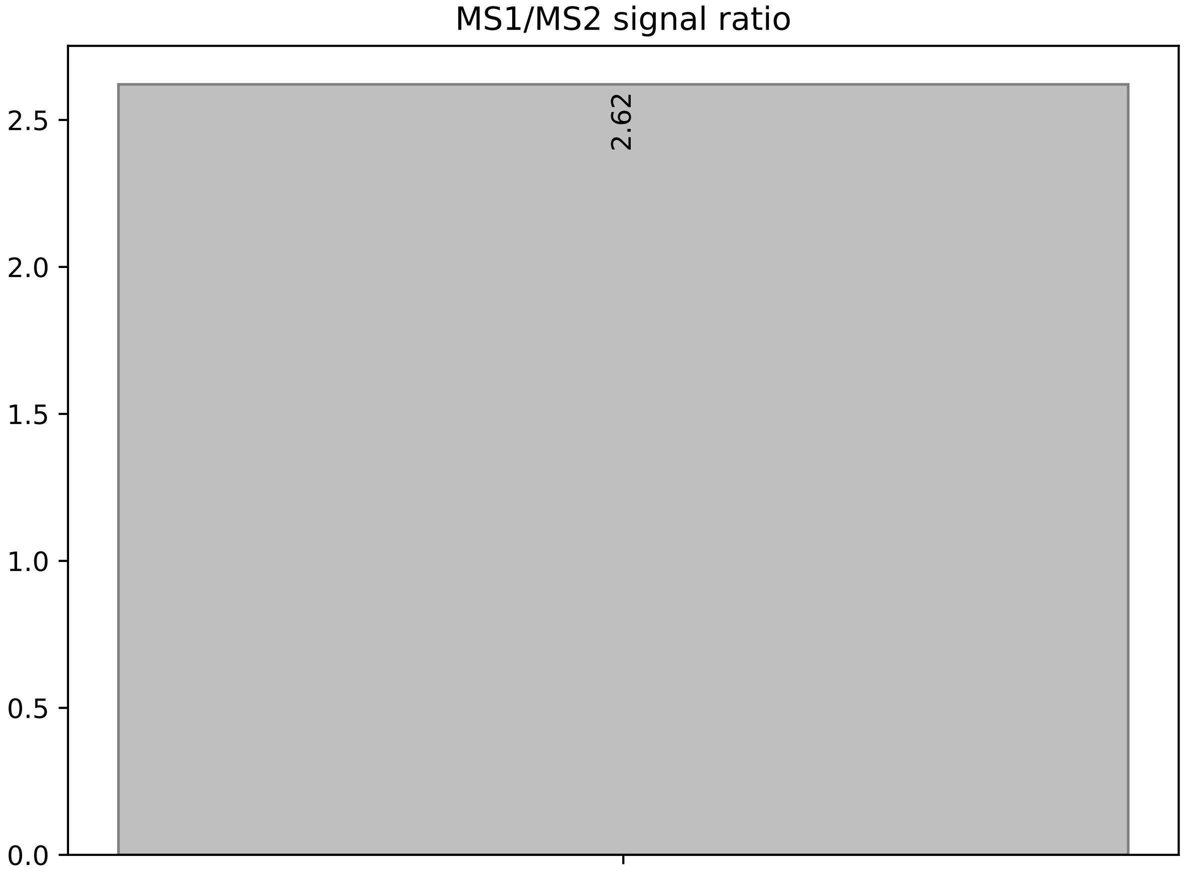
MS1/MS2 signal ratio
2.62
0.0
0.5
1.0
1.5
2.0
2.5

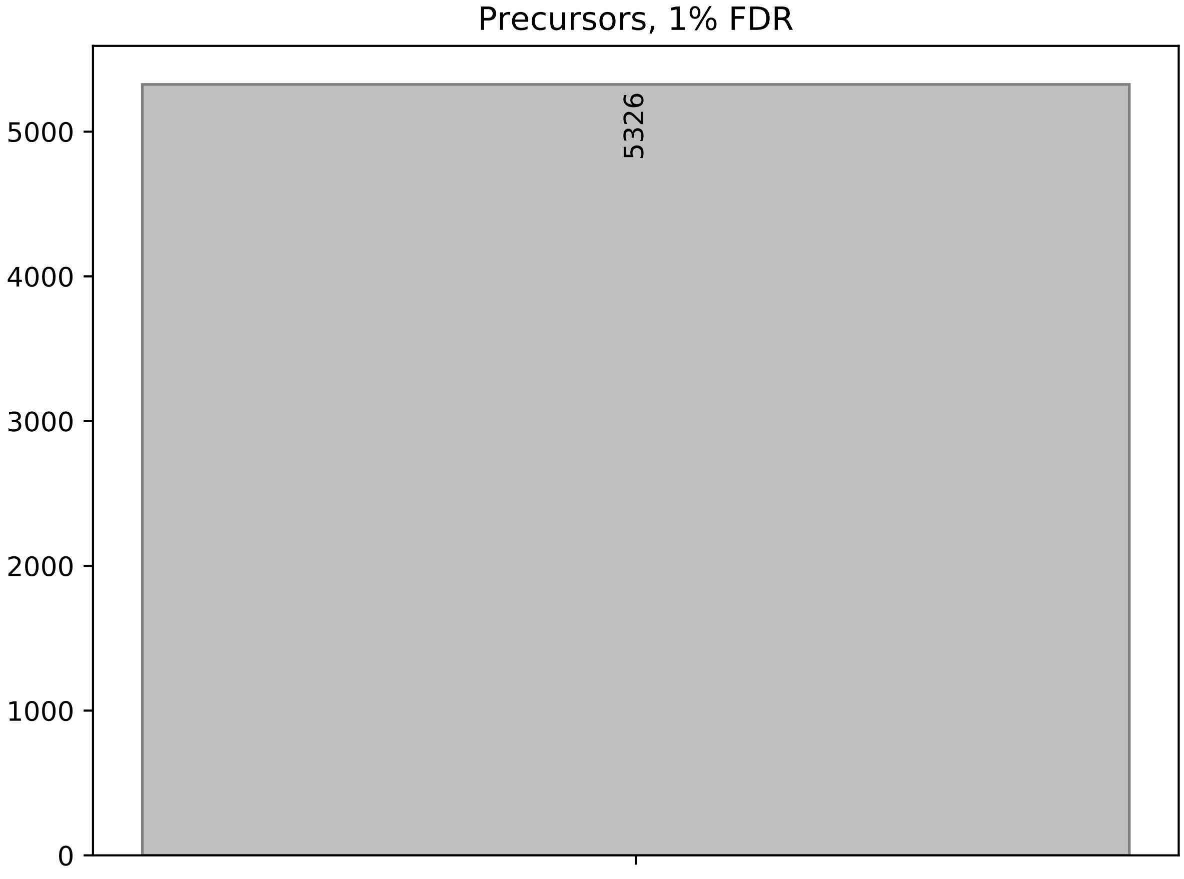
Precursors, 1% FDR
5326
0
1000
2000
3000
4000
5000

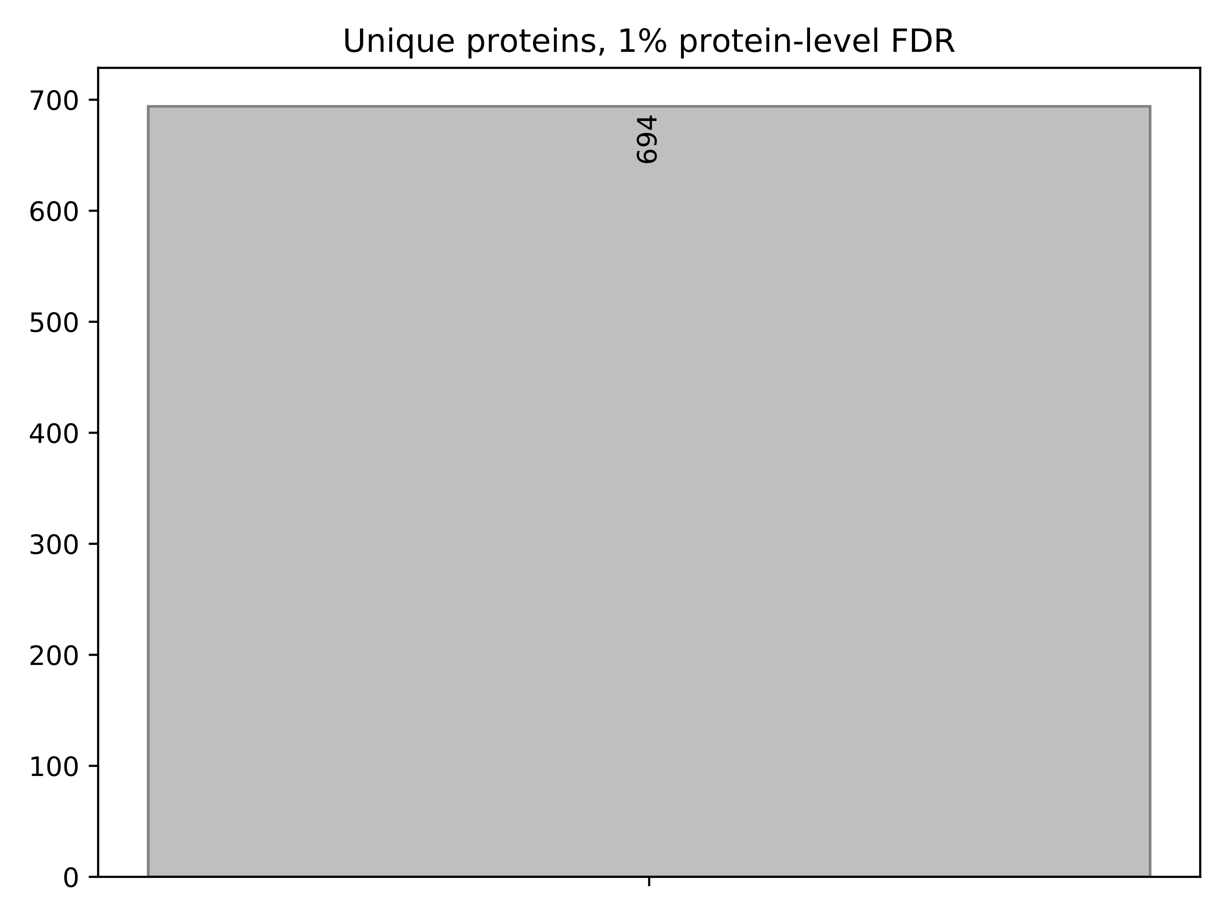

Unique proteins, 1% protein-level FDR
700
600
500
400
300
200
100
0
694

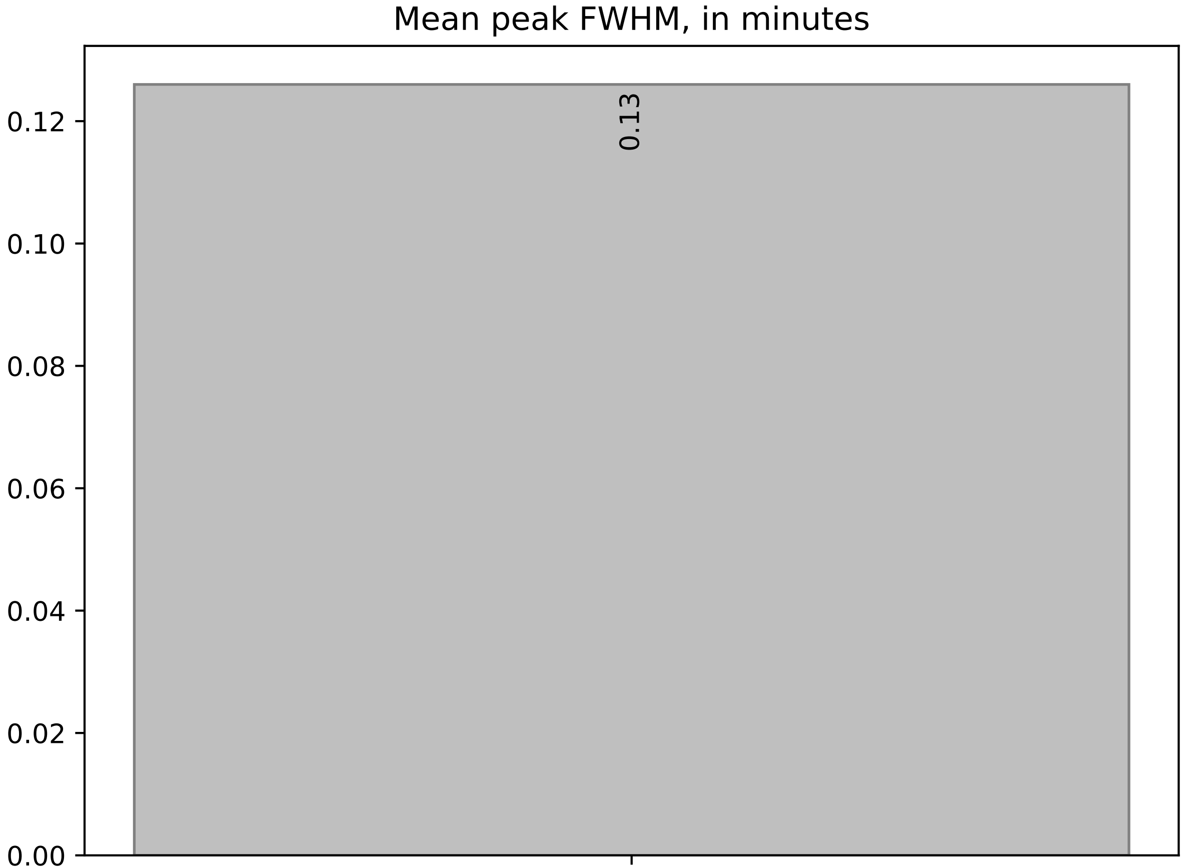
Mean peak FWHM, in minutes
0.13
0.12
0.10
0.08
0.06
0.04
0.02
0.00

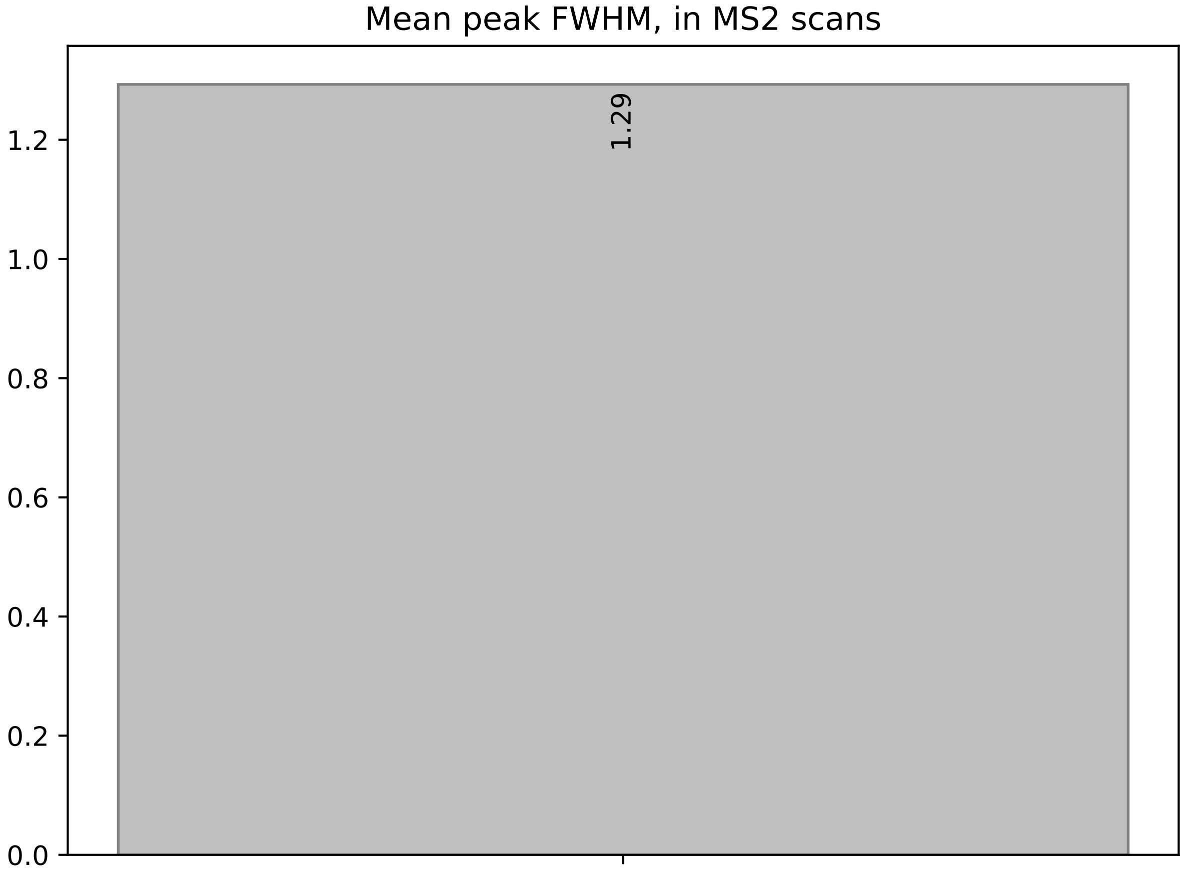

Mean peak FWHM, in MS2 scans
1.29
0.0
0.2
0.4
0.6
0.8
1.0
1.2

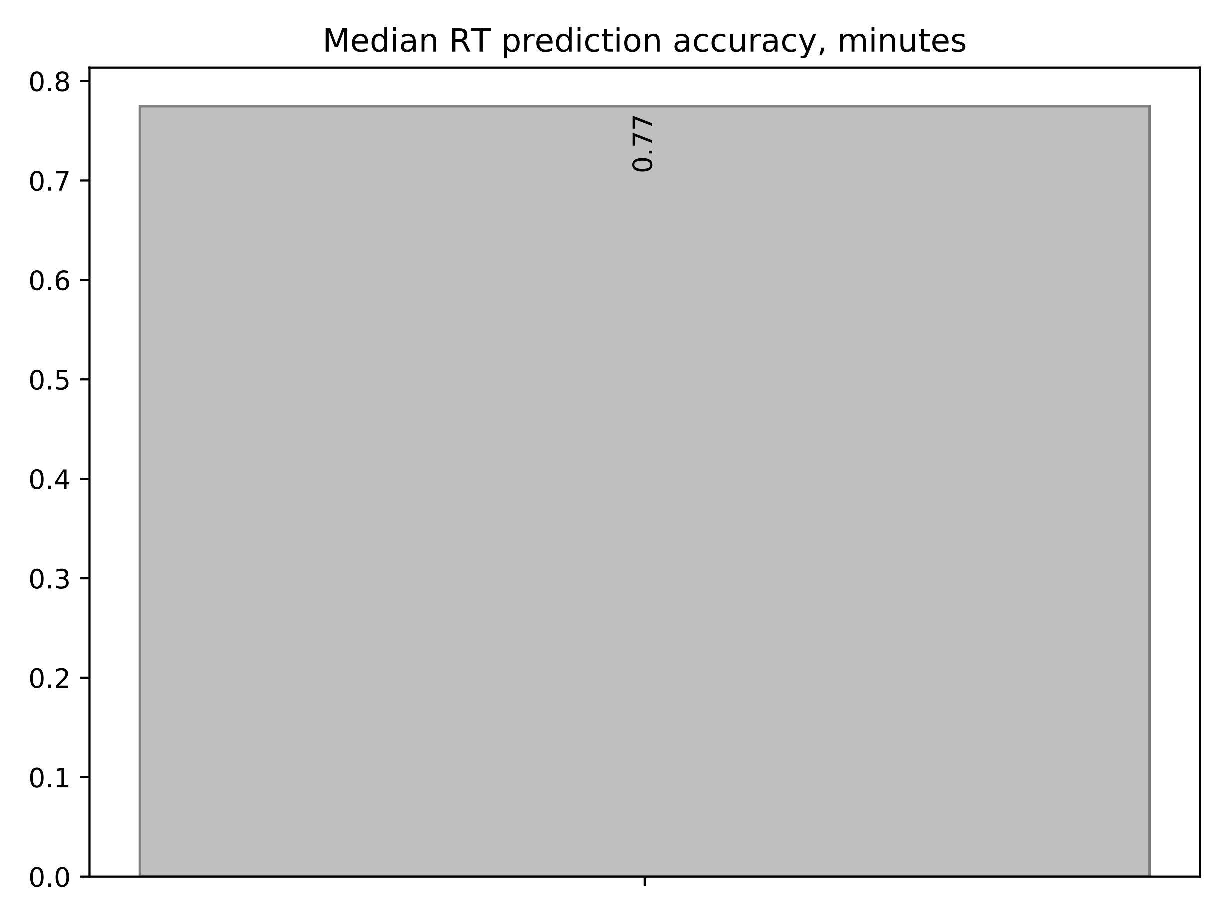
Median RT prediction accuracy, minutes
0.8
0.7
0.6
0.5
0.4
0.3
0.2
0.1
0.0
0.77

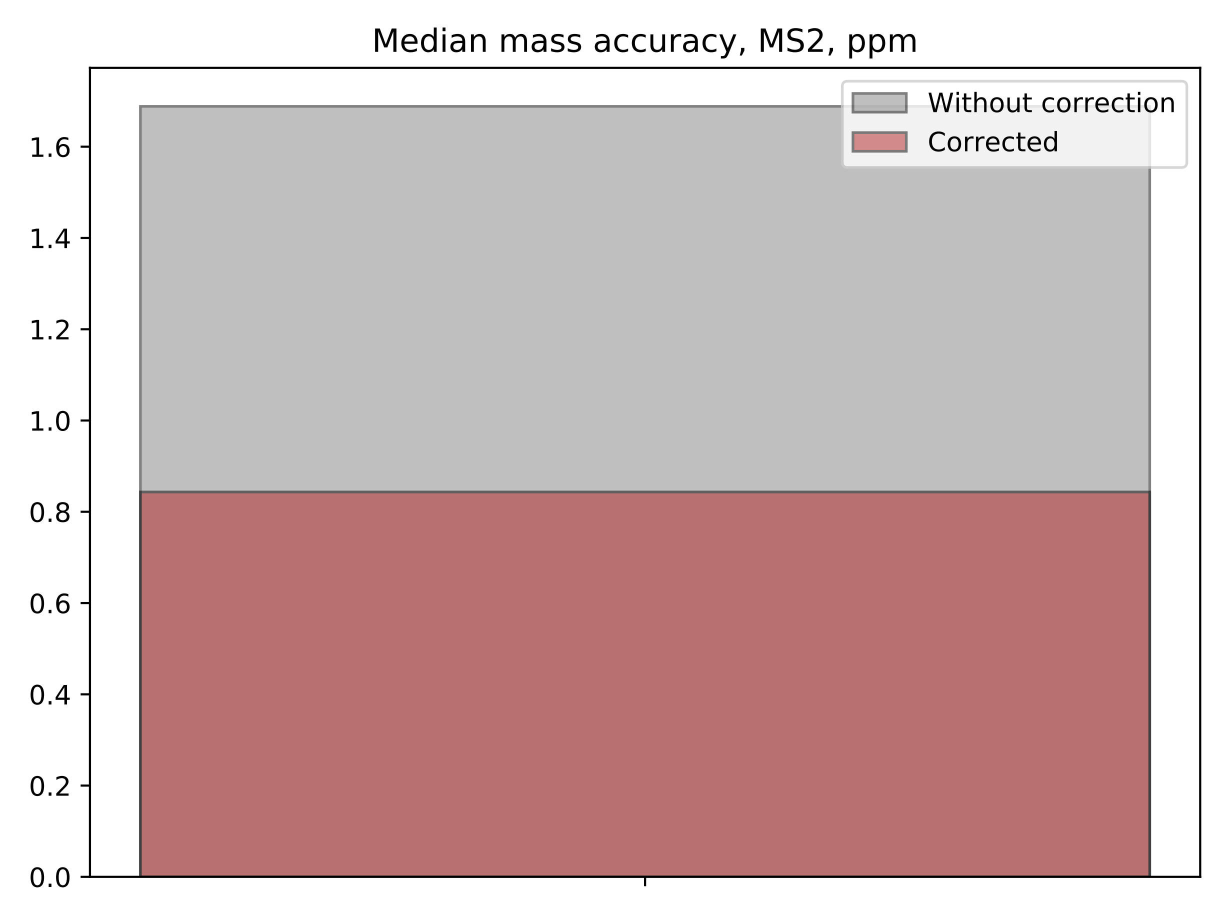
Median mass accuracy, MS2, ppm
Without correction
Corrected
0.0
0.2
0.4
0.6
0.8
1.0
1.2
1.4
1.6

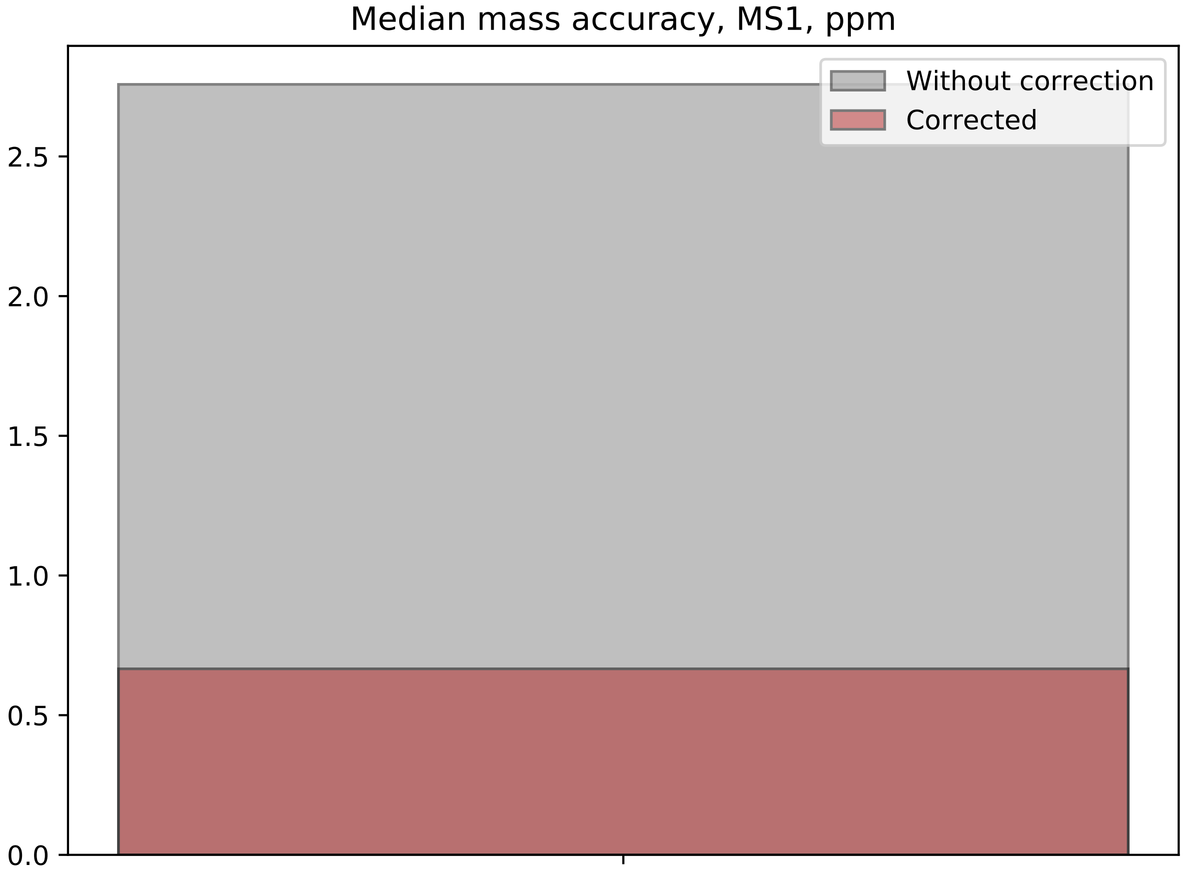
Median mass accuracy, MS1, ppm
Without correction
Corrected
0.0
0.5
1.0
1.5
2.0
2.5

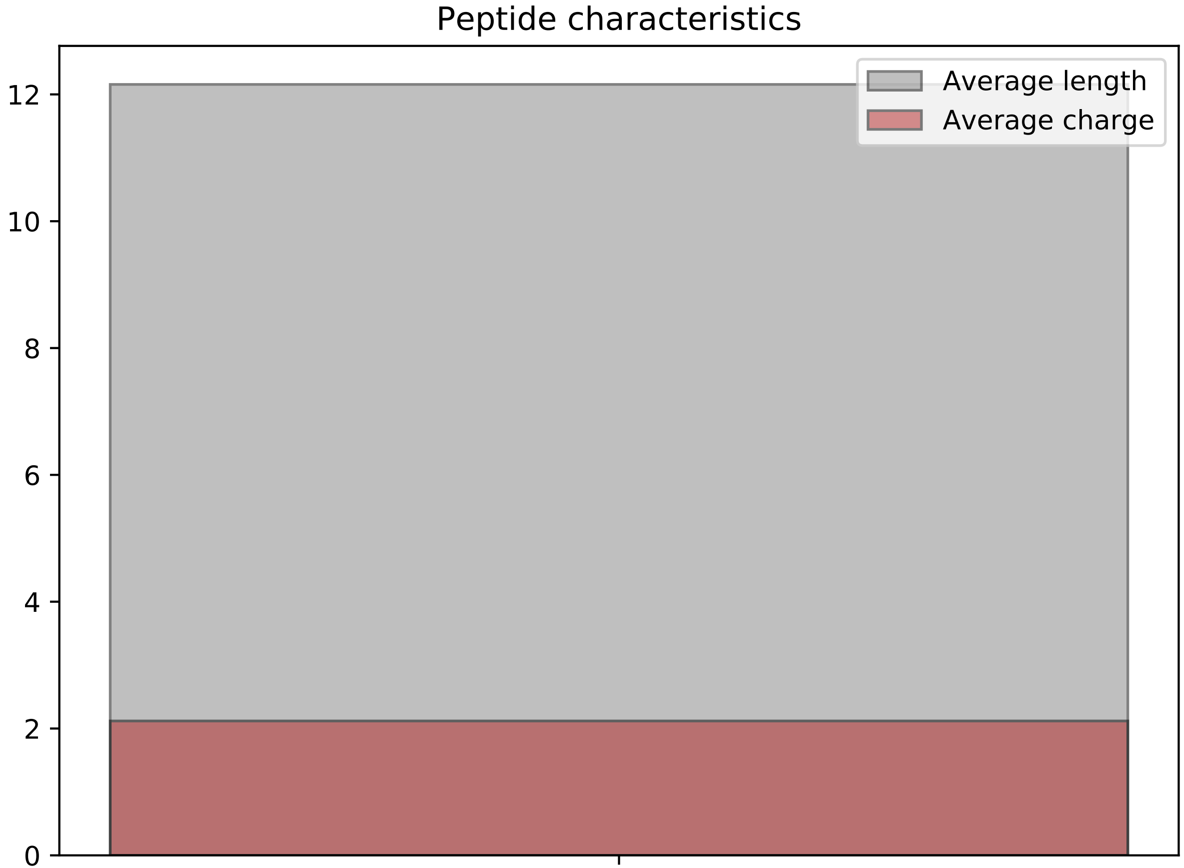

Peptide characteristics
Average length
Average charge
0
2
4
6
8
10
12

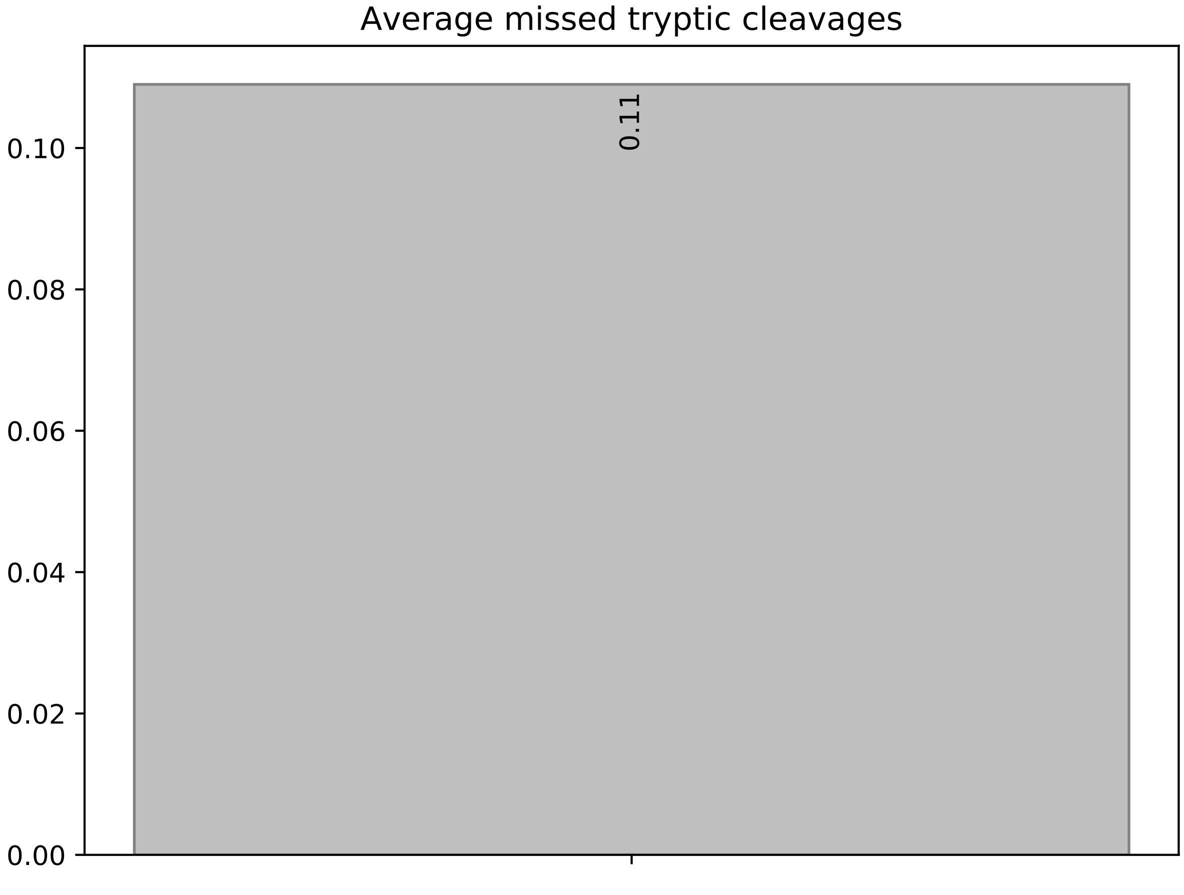
Average missed tryptic cleavages
0.11
0.10
0.08
0.06
0.04
0.02
0.00
